# Alcohol drinking patterns have a positive association with cognitive function among older people: a cross-sectional study

**DOI:** 10.1186/s12877-022-02852-8

**Published:** 2022-02-28

**Authors:** Yuya Akagi, Mai Kabayama, Yasuyuki Gondo, Yukie Masui, Saori Yasumoto, Nonglak Klinpudtan, Werayuth Srithumsuk, Kayo Godai, Kazunori Ikebe, Hiroshi Akasaka, Serina Yokoyama, Yoichi Nozato, Yoichi Takami, Yasushi Takeya, Koichi Yamamoto, Ken Sugimoto, Yasumichi Arai, Hiroki Inagaki, Tatsuro Ishizaki, Hiromi Rakugi, Kei Kamide

**Affiliations:** 1 Division of Health Sciences, Osaka University Graduate School of Medicine, Osaka, Japan; 2 Graduate School of Human Sciences, Osaka University, Osaka, Japan; 3 Tokyo Metropolitan Geriatric Hospital and Institute of Gerontology, Tokyo, Japan; 4 Department of Prosthodontics, Gerodontology and Oral Rehabilitation, Osaka University Graduate School of Dentistry, Osaka, Japan; 5 Department of Geriatric and General Medicine, Osaka University Graduate School of Medicine, Osaka, Japan; 6 Department of General and Geriatric Medicine, Kawasaki Medical University, Okayama, Japan; 7 Center for Supercentenarian Medical Research, Keio University School of Medicine, Tokyo, Japan

**Keywords:** Alcohol drinking patterns, Cognitive function, Older people

## Abstract

**Background:**

The relationship between moderate alcohol drinking or other alcohol drinking patterns such as frequency, beverage type, and situation of drinking and cognitive function is not sufficiently clear in older people. The purpose of this study was to investigate the association between alcohol drinking patterns and cognitive function in community-dwelling Japanese people aged 75 and over.

**Methods:**

This study was a cross-sectional design based on a prospective cohort study called the SONIC study. Subjects were older people aged 75-77 or 85-87 who voluntarily participated in 2016-2017. Drinking information was collected for daily drinking frequency, daily drinking intake, beverage type, and non-daily drinking opportunity. Cognitive function was measured using the Japanese version of the Montreal Cognitive Assessment (MoCA-J). Other potential confounding factors evaluated were age, sex, medical factors, and psychosocial factors. An analysis of covariance was performed to evaluate the MoCA-J score relative to drinking frequency or alcohol intake. Multiple regression analysis was performed to investigate the association between beverage type or non-daily drinking opportunity and the MoCA-J score.

**Results:**

The final number of participants analyzed was 1,226. The MoCA-J score for participants who reported drinking alcohol 1–6 days/week was significantly higher than that for those who reported drinking none or every day. No significant difference in the MoCA-J score was observed relative to daily alcohol intake. In terms of beverage type, wine was associated positively with the MoCA-J score. Non-daily drinking opportunity was also associated positively with the MoCA-J score.

**Conclusions:**

Moderate-frequency drinking, wine consumption, and non-daily drinking opportunities were associated with higher cognitive function in community-dwelling Japanese aged 75 and over. Further longitudinal studies are needed to clarify the causal relationships.

**Supplementary Information:**

The online version contains supplementary material available at 10.1186/s12877-022-02852-8.

## Background

Dementia, which is especially common among older people, affects about 50 million people worldwide and is a major public health problem [1, 2]. Because dementia is caused by irreversible neurological changes and there is currently no effective treatment, it is important to prevent cognitive decline before dementia develops. Therefore, it is necessary to identify factors related to cognitive decline and to implement preventive measures to maintain cognitive function [3].

Excessive drinking is commonly cited as a risk factor for dementia and cognitive decline [4, 5]. However, because previous studies often targeted the middle-aged or young–old in general, the relationship between drinking patterns and cognitive function in people aged 75 and over is not sufficiently clear [4–11]. In particular, interventions for nutrition in older patients are switching to those aimed at preventing frailty rather than those targeting metabolic syndromes [12]. Moderate drinking was reported to have a positive effect on good nutrition and preventive effect for cardiovascular diseases [13, 14]. It was also reported that maintaining good nutrition and preventing cardiovascular diseases contribute to preserving cognitive function [15, 16]. Therefore, following these pathways, there is a possibility that moderate alcohol drinking has a positive effect on cognitive function. Given that there is inconsistent data investigating the relationship between moderate drinking and cognitive function, determination of the overall effect of alcohol use is required, especially for older population [4, 6–8]. Because previous studies generally focused on the volume of alcohol consumed, careful consideration of alcohol drinking patterns, such as the frequency of drinking, the type of beverage, and the situation of drinking, could be used to elucidate these relationships. Drinking behavior (situation of drinking) may include elements of social participation, which are important protective factors for cognitive function in older people [17, 18]. However, there are few studies on the relationship between alcohol drinking and cognitive function in older people, which consider detailed alcohol drinking patterns and social factors. It is unclear whether alcohol drinking is a confounding factor between social relationships and cognitive function, or whether it is before or after social factors in the pathway hypothesis, and it is necessary to examine it from both sides. Several protective associations between alcoholic beverage type and cognitive function were reported, especially for moderate amounts of wine [10, 11, 19]. However, these studies concluded that these effects were due to the influence from a Mediterranean diet and few studies targeted Asian populations, such as the Japanese population, who generally have a relatively weak alcohol tolerance and different food culture. In addition, it is not clear how the beverage types that are often consumed in Japan, such as sake and Japanese spirits, affect cognitive function.

Therefore, the purpose of this study was to investigate the association between drinking patterns and cognitive function in community-dwelling Japanese people aged 75 and over to provide evidence to contribute to the optimal drinking recommendations in this super-aged society.

## Methods

### Study participants

This study was a cross-sectional design based on a prospective cohort study called the SONIC (Septuagenarians, Octogenarians, Nonagenarians, and Investigation with Centenarians) study, an ongoing study since 2010 with follow-ups every 3 years [20]. The eligibility criteria for the study were that the participants aged 75–77 years (76-aged group) or aged 85–87 years (86-aged group) and that they participated in 2016–2017. Overall, 1,335 individuals participated, and they were grouped based upon age: 846 people in the 76-aged group (459 women and 387 men) and 489 people in the 86-aged group (240 women and 249 men). We excluded 109 participants who had no alcohol drinking information. 95 people of the excluded participants completed door-to-door or mail surveys instead of on-site surveys, but we could not collect medical data, including alcohol drinking information. The other 14 people participated in on-site surveys, but we were unable to collect valid drinking information. The SONIC study protocol was approved by the institutional review boards of Osaka University Graduate School of Medicine, Dentistry, and Human Sciences, and the Tokyo Metropolitan Institute of Gerontology (approval numbers 266, H22-E9, 22 018, and 38, respectively). Informed consent was obtained from all participants.

### Measurements for alcohol drinking patterns

The drinking patterns, such as daily drinking frequency, daily alcohol intake, beverage type, and the situation of drinking: non-daily drinking opportunity, were collected by doctors, nurses, public health nurses, and dietitians. The daily drinking frequency was collected as follows: “None”, “<1 day in 2–3 months”, “1 day in 2–3 months”, “1 day/month”, “2–3 days/month”, “1 day/week”, “2 days/week”, “3 days/week”, “4 days/week”, “5 days/week”, “6 days/week”, and “Every day”. The data were then classified into four categories: “None”, “<1 day/week”, “1–6 days/week”, and “Every day”. Daily alcohol intake was calculated using a measuring cup after considering the beverage type. The formula for calculating alcohol intake was: “alcohol concentration (%) × amount (mL) × density (0.8 kg/m^3^)”. The alcohol concentration of each beverage was set at 5% for beer, 25% for Japanese spirits, 15% for sake, 12% for wine, and 42% for whiskey. Daily alcohol intake was classified into the following categories: “None (0 g)”, “Moderate (>0 g and <40 g)”, “Moderate to Excessive (≥40 g and <60 g)”, and “Excessive (≥60 g)” for men. For women, the threshold values used were half as high as those used for men [21]. We collected the data of the situation of drinking as non-daily drinking opportunity that was different from daily drinking, such as gatherings with relatives at the end of the year, dining with close friends at restaurants, and parties.

### Evaluation of cognitive function

Cognitive function was evaluated using the Japanese version of the Montreal Cognitive Assessment (MoCA-J), which was administered by trained gerontologists and psychologists. The MoCA-J, which is based on the MoCA, is a tool for sensitively detecting mild cognitive impairment in older Japanese people, and the score was evaluated from 0 to 30 and adjusted for years of education (1 point added for 12 years or less) [22, 23]. A higher score indicated a higher cognitive function.

### Assessment of other potential confounders

Medical factors were collected from interviews and blood samples, which were obtained by doctors, nurses, public health nurses, and dietitians. Smoking was evaluated as currently smoking or not. Stroke was assessed as positive if there was previous medical documentation. Hypertension was defined as positive if an individual had a systolic blood pressure ≥140 mmHg, had a diastolic blood pressure ≥90 mmHg, or was receiving antihypertensive treatment [24]. Diabetes mellitus was defined as positive if an individual had a HbA1c ≥6.5%, had a casual blood glucose concentration ≥200 mg/dL, or was taking medications for diabetes mellitus [25]. Dyslipidemia was defined as positive if an individual had a non–high-density lipoprotein cholesterol ≥170 mg/dL, had a high-density lipoprotein cholesterol <40 mg/dL, had a triglyceride level ≥150 mg/dL, or was taking medications for dyslipidemia [26, 27]. Atherosclerosis was evaluated using the maximum carotid intima-media thickness (Max-CIMT). The Max-CIMT was the maximum value of the left and right CIMT measured using ultrasonic echo (GE LOGIQ book XP; GE Healthcare, Tokyo, Japan), and atherosclerosis was evaluated as Max-CIMT ≥1.1 mm [28].

Psychological and social factors were collected by gerontologists and psychologists. Mental health was measured using the Japanese version of the WHO Five Well-Being Index (WHO-5-J), and a score of 13 or higher was evaluated as good [29].

Social factors were evaluated, including living alone (or living with someone else), frequency of going out (<1 time/week, 1–2 times/week, 3–4 times/week, 5–6 times/week, and every day), education (≤9 years, 10–12 years, and ≥13 years), and economic status (not satisfied, neutral, and satisfied).

### Statistical analysis

Descriptive data are shown as a percentage (%) or mean ± standard deviation (SD). For comparisons between characteristics, chi-square test or Fisher’s exact test were used for categorical variables, and one-way analysis of variance (ANOVA) or *t*-test were performed for continuous variables. To evaluate the average MoCA-J score relative to daily drinking frequency or daily alcohol intake, analysis of covariance (ANCOVA) adjusted for potential confounders was performed. Multiple comparison tests for the categorical variables treated as dummy variables were used with the Tukey-Kramer tests for *post hoc* analysis. Univariate regression analysis was performed to examine the relationship between beverage type or non-daily drinking opportunity and the MoCA-J scores. Multiple regression analysis adjusted for potential confounders was performed using the drinking pattern variables. In each multivariable analysis, the interaction with sex was examined concerning the drinking pattern variable and the MoCA-J score. *p*-values <0.05 were considered statistically significant. All analyses were performed using SPSS Statistics version 27 (IBM Japan, Tokyo, Japan).

## Results

### Characteristics of the study population relative to daily drinking frequency

The number of participants analyzed in this study was 1,226. Table 1 shows the characteristics of the population relative to daily drinking frequency. The proportion of daily drinking frequency from highest to lowest was: none (55.5%), every day (25.7%), 1–6 days/week (13.5%), and <1 day/week (5.4%) (Additional file 1: Figure S1  shows the details for daily drinking frequency). The proportion of daily alcohol intake from highest to lowest was: none (55.8%), moderate (34.8%), moderate to excessive (5.8%), and excessive (3.6%). About half of the participants (50.1%) reported non-daily drinking opportunities. The proportion of beverage type from highest to lowest was: beer (24.3%), Japanese spirits (13.1%), sake (10.8%), wine (4.4%), and whiskey (2.6%). Some individuals consumed multiple types of alcohol. When we evaluated the subject characteristics relative to daily drinking frequency, significant differences were observed for age group, sex, daily alcohol intake, non-daily drinking opportunity, all beverage types, hypertension, dyslipidemia, living alone, frequency of going out, education, and economic status. The mean overall MoCA-J score was 22.7, and there were significant differences in daily drinking frequency.

**Table 1:** Comparison of characteristics relative to daily drinking frequency

			Daily drinking frequency (per week)								
	Total		None		<1 day		1-6 days		Every day		
Characteristic	(*n* = 1226)		(*n* = 680)		(*n* = 66)		(*n* = 165)		(*n* = 315)		*p*-value
Age: 76 aged group, n (%)	743	(60.6)	385	(56.6)	43	(65.2)	114	(69.1)	201	(63.8)	<0.01
Sex: Men, n (%)	594	(48.5)	220	(32.4)	29	(43.9)	94	(57.0)	251	(79.7)	<0.01
Daily alcohol intake, n (%)											<0.01
None	680	(55.8)	680	(100.0)	0	(0.0)	0	(0.0)	0	(0.0)	
Moderate	424	(34.8)	0	(0.0)	58	(93.5)	136	(82.9)	230	(73.5)	
Moderate to excessive	71	(5.8)	0	(0.0)	3	(4.8)	17	(10.4)	51	(16.3)	
Excessive	44	(3.6)	0	(0.0)	1	(1.6)	11	(6.7)	32	(10.2)	
Beverage type, n (%)											
Beer	298	(24.3)	0	(0.0)	40	(60.6)	86	(52.1)	172	(54.6)	<0.01
Japanese spirits	161	(13.1)	0	(0.0)	5	(7.6)	42	(25.5)	114	(36.2)	<0.01
Sake	132	(10.8)	0	(0.0)	9	(13.6)	36	(21.8)	87	(27.6)	<0.01
Wine	54	(4.4)	0	(0.0)	10	(15.2)	24	(14.5)	20	(6.3)	<0.01
Whisky	32	(2.6)	0	(0.0)	1	(1.5)	7	(4.2)	24	(7.6)	<0.01^a^
Non-daily drinking opportunity, n (%)	606	(50.1)	219	(32.5)	41	(62.1)	117	(71.3)	229	(74.8)	<0.01
Current smoking, n (%)	77	(6.3)	35	(5.2)	3	(4.5)	10	(6.1)	29	(9.3)	0.09
Stroke, n (%)	117	(9.6)	63	(9.3)	7	(10.6)	18	(10.9)	29	(9.2)	0.91
Hypertension, n (%)	892	(73.3)	486	(72.1)	49	(75.4)	110	(67.1)	247	(78.7)	<0.05
Diabetes mellitus, n (%)	214	(18.0)	123	(18.7)	14	(22.2)	32	(19.5)	45	(14.7)	0.33
Dyslipidemia, n (%)	774	(64.4)	474	(70.9)	37	(57.8)	96	(58.9)	167	(54.8)	<0.01
Atherosclerosis, n (%)	995	(81.2)	536	(78.8)	57	(86.4)	140	(84.8)	262	(83.4)	0.10
WHO-5-J (≥ 13), n (%)	962	(78.8)	542	(79.9)	50	(76.9)	129	(78.2)	241	(77.0)	0.73
Living alone, n (%)	288	(23.7)	197	(29.3)	9	(13.6)	41	(25.0)	41	(13.1)	<0.01
Frequency of going out, n (%)											<0.05
<1 time/week	82	(6.7)	43	(6.4)	10	(15.2)	9	(5.5)	20	(6.4)	
1–2 times/week	191	(15.7)	111	(16.4)	15	(22.7)	23	(14.0)	42	(13.4)	
3–4 times/week	278	(22.8)	154	(22.8)	11	(16.7)	46	(28.0)	67	(21.3)	
5–6 times/week	228	(18.7)	135	(20.0)	12	(18.2)	28	(17.1)	53	(16.9)	
Every day	440	(36.1)	232	(34.4)	18	(27.3)	58	(35.4)	132	(42.0)	
Education, n (%)											<0.05
≤9 years	294	(24.0)	169	(24.9)	13	(19.7)	32	(19.4)	80	(25.4)	
10–12 years	576	(47.1)	340	(50.1)	29	(43.9)	78	(47.3)	129	(41.0)	
≥13 years	354	(28.9)	169	(24.9)	24	(36.4)	55	(33.3)	106	(33.7)	
Economic status, n (%)											0.30
Not satisfied	220	(18.0)	129	(19.1)	10	(15.2)	37	(22.6)	44	(14.0)	
Neutral	748	(61.4)	405	(60.0)	41	(62.1)	98	(59.8)	204	(65.0)	
Satisfied	251	(20.6)	141	(20.9)	15	(22.7)	29	(17.7)	66	(21.0)	
MoCA-J score, mean (SD)	22.7	(3.9)	22.6	(4.0)	23.2	(3.6)	23.6	(3.7)	22.5	(3.7)	<0.05

Notes: 76 and 86 aged groups included subjects 75–77 and 85–87 years old, respectively. The criteria for alcohol intake were defined as follows. For men, “Moderate” was >0 g and <40 g, “Moderate to Excessive” was ≥40 g and <60 g, and “Excessive” was ≥60 g. For women, the threshold values used were half as high as those used for men

Abbreviations: *SD* standard deviation; *WHO-5-J* Japanese version of the WHO Five Well-Being Index; *MoCA-J* Japanese version of the Montreal Cognitive Assessment

*p*-values were based on chi-square tests for categorical variables and analysis of variance for continuous variables.

^a^*p*-values were based on Fisher’s Exact test

### Comparison of MoCA-J scores relative to daily drinking frequency

**Fig. 1:**
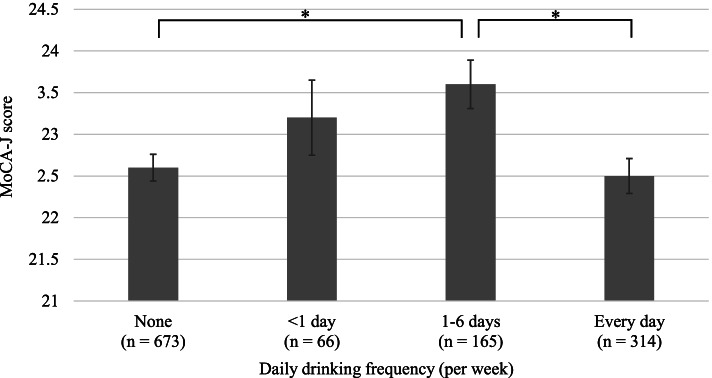
Comparison of the MoCA-J score relative to daily drinking frequency using the analysis of covariance (ANCOVA) after adjusting for age group, sex, daily alcohol intake, beer consumption, Japanese spirits consumption, sake consumption, wine consumption, whisky consumption, non-daily drinking opportunity, current smoking, stroke, hypertension, diabetes mellitus, dyslipidemia, atherosclerosis, WHO-5-J, living alone, frequency of going out, and economic status. Multiple comparison tests for the categorical variables were used with the Tukey-Kramer tests for *post hoc*analysis. The error bars represent the standard error

Figure 1 shows the results from ANCOVA analysis, where we evaluated the MoCA-J scores relative to daily drinking frequency. After adjusting for age group, sex, daily alcohol intake, beer consumption, Japanese spirits consumption, sake consumption, wine consumption, whisky consumption, non-daily drinking opportunity, current smoking, stroke, hypertension, diabetes mellitus, dyslipidemia, atherosclerosis, WHO-5-J, living alone, frequency of going out, and economic status, the MoCA-J score for individuals consuming alcohol 1–6 days/week was significantly higher than those for none or every day in multiple comparisons. No significant difference was observed relative to the other multiple comparison combinations.

### Comparison of MoCA-J scores relative to daily alcohol intake

**Fig. 2:**
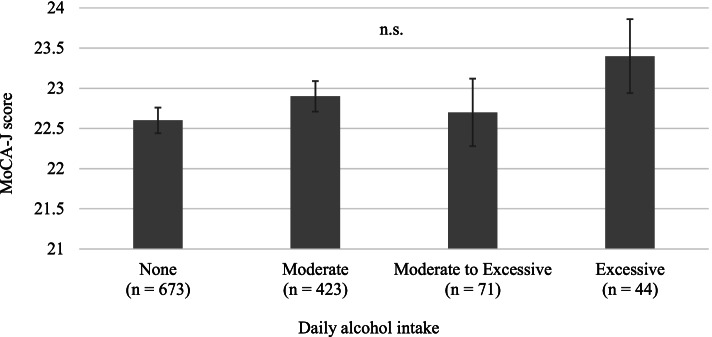
Comparison of the MoCA-J score relative to daily drinking frequency using the analysis of covariance (ANCOVA) after adjusting for age group, sex, daily alcohol intake, beer consumption, Japanese spirits consumption, sake consumption, wine consumption, whisky consumption, non-daily drinking opportunity, current smoking, stroke, hypertension, diabetes mellitus, dyslipidemia, atherosclerosis, WHO-5-J, living alone, frequency of going out, and economic status. Multiple comparison tests for the categorical variables were used with the Tukey-Kramer tests for *post hoc*analysis. The error bars represent the standard error

Figure 2 shows the results from ANCOVA analysis, where we evaluated the MoCA-J scores relative to daily alcohol intake (Additional file 2: Table S1 shows the comparison of characteristics relative to daily alcohol intake). After adjusting for age group, sex, daily drinking frequency, beer consumption, Japanese spirits consumption, sake consumption, wine consumption, whisky consumption, non-daily drinking opportunity, current smoking, stroke, hypertension, diabetes mellitus, dyslipidemia, atherosclerosis, WHO-5-J, living alone, frequency of going out, and economic status, there was no significant difference between all combinations. 

### Relationship between drinking patterns and MoCA-J scores

Table 2 shows the results of the relationship between drinking patterns and the MoCA-J scores. For univariate analysis, the significant positive correlations with the MoCA-J scores were drinking frequency (1-6 days/week for every day), wine consumption, non-daily drinking frequency, sex, and frequency of going out, and the significant negative correlations were age group, diabetes mellitus, atherosclerosis, and WHO-5-J. For multiple regression analysis adjusted for all the potential confounders (daily drinking frequency, daily alcohol intake, beer consumption, Japanese spirits consumption, sake consumption, whisky consumption, age group, sex, smoking status, stroke, hypertension, diabetes mellitus, dyslipidemia, atherosclerosis, WHO-5-J, living alone, frequency of going out, and economic status), wine consumption (β = 0.09, *p*<0.01) and non-daily drinking opportunity (β = 0.09, *p*<0.01) significantly positively correlated with the MoCA-J scores (Additional files 3 and 4: Tables S2 and S3 show the comparison of characteristics relative to wine and non-daily drinking opportunity, respectively).

**Table 2:** Factors influencing drinking patterns associated with MoCA-J score, determined using multiple regression analysis

	Univariate				Multivariable			
	B (95% CI)		β		B (95% CI)		β	
Daily drinking frequency (reference = every day)								
None/week	0.04	(-0.48, 0.57)		0.01	0.46	(-0.61, 1.53)		0.06
<1 day/week	0.72	(-0.32, 1.75)		0.04	0.68	(-0.40, 1.75)		0.04
1-6 days/week	1.06	(0.33, 1.80)		0.09**	0.83	(0.10, 1.56)		0.08*
Daily alcohol intake^a^	0.23	(-0.06, 0.52)		0.04	-0.47	(-1.04, 0.11)	-	0.09
Beverage type (1 = yes)								
Beer	0.45	(-0.07, 0.96)		0.05	0.41	(-0.31, 1.14)		0.05
Japanese spirits	0.44	(-0.21, 1.09)		0.04	1.17	(-0.32, 2.03)		0.08
Sake	-0.41	(-1.11, 0.30)	-	0.03	0.41	(-0.52, 1.19)		0.03
Wine	1.80	(0.74, 2.87)		0.10**	1.58	(0.48, 2.68)		0.09**
Whisky	0.77	(-0.61, 2.14)		0.03	0.97	(-0.39, 2.34)		0.04
Non-daily drinking opportunity (1 = yes)	0.90	(0.46, 1.35)		0.12**	0.70	(0.23, 1.16)		0.09**
(Adjustment variables)								
Age (1 = 86 aged group)	-2.22	(-2.66, -1.80)	-	0.28**	-1.82	(-2.30, -1.34)	-	0.23**
Sex (1 = women)	0.83	(0.39, 1.27)		0.11**	0.85	(0.34, 1.35)		0.11**
Current smoking (1 = yes)	-0.11	(-1.02, 0.80)	-	0.01	-0.08	(-0.99, 0.83)	-	0.01
Stroke (1 = yes)	-0.34	(-1.08, 0.41)	-	0.03	0.13	(-0.60, 0.86)		0.01
Hypertension (1 = yes)	-0.28	(-0.77, 0.22)	-	0.03	0.06	(-0.45, 0.56)	-	0.01
Diabetes mellitus (1 = yes)	-0.62	(-1.19, -0.04)	-	0.06*	-0.47	(-1.03, 0.10)	-	0.05
Dyslipidemia (1 = yes)	0.34	(-0.12, 0.80)		0.04	0.30	(-0.15, 0.75)		0.04
Atherosclerosis (1 = yes)	-0.87	(-1.44, -0.31)	-	0.09**	-0.04	(-0.61, 0.52)	-	0.00
WHO-5-J (1 = <13)	-0.77	(-1.30, -0.23)	-	0.08**	-0.42	(-0.95, 0.11)	-	0.04
Living alone (1 = yes)	-0.26	(-0.78, 0.26)	-	0.03	-0.22	(-0.75, 0.31)	-	0.02
Frequency of going out^a^	0.54	(0.38, 0.71)		0.18**	0.38	(0.21, 0.55)		0.13**
Economic status^a^	0.26	(-0.10, 0.61)		0.04	0.35	(0.02, 0.70)		0.05*
Adjusted R^2^								0.12

^a^Larger numbers were assigned to those with higher frequency, alcohol intake and status

Abbreviations: *B* partial regression coefficient; *CI* confidence interval; *β* standardized partial regression coefficient; *WHO-5-J* Japanese version of the WHO Five Well-Being Index; *MoCA-J* Japanese version of the Montreal Cognitive Assessment

*p<0.05 and **p<0.01

There were no sex interactions (*p*>0.05 in each analyses) in the associations between all drinking patterns and cognitive function (Additional file 5: Table S4 shows the comparison of characteristics relative to sex).

## Discussion

Our study examined the association between alcohol drinking patterns, such as drinking frequency, alcohol intake, beverage type, and non-daily drinking opportunity, and cognitive function in community-dwelling Japanese people aged 75 and over. The main findings of this study are as follows: (1) For drinking frequency, the cognitive function of moderate-frequency drinkers, such as those who drank 1–6 days/week, was higher than those for individuals who reported their consumption as none or every day; (2) For beverage type, the cognitive function of wine drinkers was higher than that of non-drinkers; and (3) For drinking opportunity, the cognitive function of those who engaged in non-daily drinking opportunities was higher than that of those who did not. Daily alcohol intake was not associated with increased cognitive function.

Few studies have reported an association between cognitive function and drinking frequency among the alcohol drinking patterns of the older population [30]. This is the first study that investigated drinking frequency and cognitive function for a population of individuals ≥75 years old. A new finding in our study was the independent association of alcohol drinking frequency; that is, moderate-frequency drinkers had higher cognitive function than non- or everyday drinkers (Fig. 1). One possible explanation for this relationship may be an effect from drinking behavior, which is associated with a variety of social factors such as promoting social activities [17]. This is an important protective factor for the cognitive decline of older people [31]. We speculate that these social aspects, along with drinking behavior, positively influenced the higher cognitive function results for moderate-frequency drinkers (1–6 days/week) relative to non-drinkers. In this study, we found a relationship between drinking frequency and cognitive function after adjusting for social factors such as living alone, frequency of going out, and economic status. The pathway hypothesis of these results is that at least the drinking frequency is associated with cognitive function independently of other social factors. In other words, it is possible that behaviors such as going out are not intermediate factors, and behavioral patterns associated with alcohol drinking affect cognitive function. However, in reality, the relationship between alcohol consumption, social factors, and cognitive function is very complicated, because social factors may include not only the factors used in this study but also various other factors such as leisure activities [32]. Therefore, drinking frequency may be a proxy variable for social factors. It is also possible that social factors are an intermediate factor between drinking frequency and cognitive function. By contrast, lower cognitive function was found in everyday drinkers. This could be due to the various adverse effects from daily, continuous alcohol exposure [33–35].

Excessive drinking has a negative effect on dementia and cognitive function [4, 5]. However, we did not observe a negative association between daily alcohol consumption and cognitive function in this study, which does not agree with the results from these previous studies (Fig. 2). Considering the characteristics of the study population including this very old population, it is possible that participants who were relatively healthy or having high health literacy could come to the survey. We speculate these survival bias and sampling bias affected these results [36]. There is also the possibility of a reversal causal effect, with healthier people drinking more. Taken together, our findings suggest that drinking frequency is an important factor that affects cognitive function in older people, but the amount of alcohol consumed is not.

For the type of alcohol consumed, we found that only wine was positively associated with cognitive function (Table 2). This result is in agreement with many previous studies that identified the protective effects of moderate wine consumption on cognitive function [10, 11, 19]. Resveratrol, a type of polyphenol in wine, may have a direct neuroprotective effect on cognitive function [37, 38]. Furthermore, we found that the proportion of highly educated people is higher in wine drinkers than in non-drinkers. This variable may have a greater impact on cognitive function, even though we adjusted the MoCA-J score based upon the level of education (Additional file 4: Table S3).

In addition, non-daily drinking opportunity was associated with higher cognitive function. Similar to the relationship between drinking frequency and cognitive function, social factors such as communication with others may have positively influenced cognitive function [17, 31]. Today, the risk of drinking is emphasized, and healthcare professionals often recommended that people decrease alcohol consumption or stop drinking overall. If drinking plays a protective role in cognitive function in older people, it may be beneficial to provide treatment and health guidance that respects their lifestyle and quality of life, rather than uniformly recommending that individuals decrease or stop drinking.

This study has several limitations. First, a survival bias effect may play a role in the association between alcohol drinking patterns and cognitive function. The subjects in this study were older people who voluntarily participated, and they tended to be healthier than the average older Japanese population because they had enough physical function to participate in a site survey. Second, drinking information was evaluated by interviews. Self-reporting methods of typical weekly alcohol intake are useful for epidemiological studies, but not perfect [39, 40]. For example, there is the possibility that the alcohol intake per week was often inconsistent, and also the recall bias may have been influenced strongly among the older population. In addition, since there was no valid classification for drinking frequency, we classified it into “None”, “<1 day/week”, “1-6 days/week”, and “Every day” based on the sensitivity analysis which explained our results reasonably considering the previous studies [33–35]. However, these cut-offs may be insufficient, so the results of this study should be interpreted in consideration of these limitations. Third, because this study was a cross-sectional study, no causal relationship could be mentioned. The association that non-drinkers had lower cognitive function than those who drink 1-6 days a week may be a reverse association that they cannot drink because they are not healthy, as mentioned in the relationship between alcohol intake and cognitive function [36]. Therefore, a longitudinal analysis is needed to clarify the causal relationship between alcohol consumption and cognitive function after adjusting for background factors.

## Conclusions

In conclusion, we observed that moderate-frequency drinking, wine consumption, and non-daily drinking opportunities were associated with higher cognitive function in community-dwelling Japanese aged 75 and over. Although the association between drinking frequency, situation of drinking, beverage type and cognitive function is new findings, the reverse association between drinking and cognitive function is often concerned. Further longitudinal studies are needed to clarify the causal relationships.

## Supplementary information

**Additional file 1:** ** Figure S1**. Details for daily drinking frequency.

**Additional file 2:** **Table S1.** Comparison of characteristics relative to daily alcohol intake.

**Additional file 3:** **Table S2.** Comparison of characteristics relative to wine consumption.

**Additional file 4:** **Table S3.** Comparison of characteristics relative to non-daily drinking opportunity.

**Additional file 5:** **Table S4.** Comparison of characteristics relative to sex.

## Data Availability

The datasets used and/or analyzed during the current study are available from the corresponding author on reasonable request.

## Abbreviations

MoCA-J: Japanese version of the Montreal Cognitive Assessment
SONIC: Septuagenarians, Octogenarians, Nonagenarians, and Investigation with Centenarians
CIMT: Carotid intima-media thickness
WHO-5-J: The Japanese version of the WHO Five Well-Being Index
SD: Standard deviation
ANOVA: Analysis of variance
ANCOVA: Analysis of covariance
B: partial regression coefficient
CI: confidence interval
β: standardized partial regression coefficient
