# Supplementary material for: Alcohol drinking patterns have a positive association with cognitive function among older people: a cross-sectional study
Source: BMC Geriatr. 2022 Feb 28;22:158. doi: 10.1186/s12877-022-02852-8 (PMC8883620; doi:10.1186/s12877-022-02852-8)
Supplement: Supplementary file 1 — Additional file 1: Figure S1. Details for daily drinking frequency. [file 12877_2022_2852_MOESM1_ESM.docx]

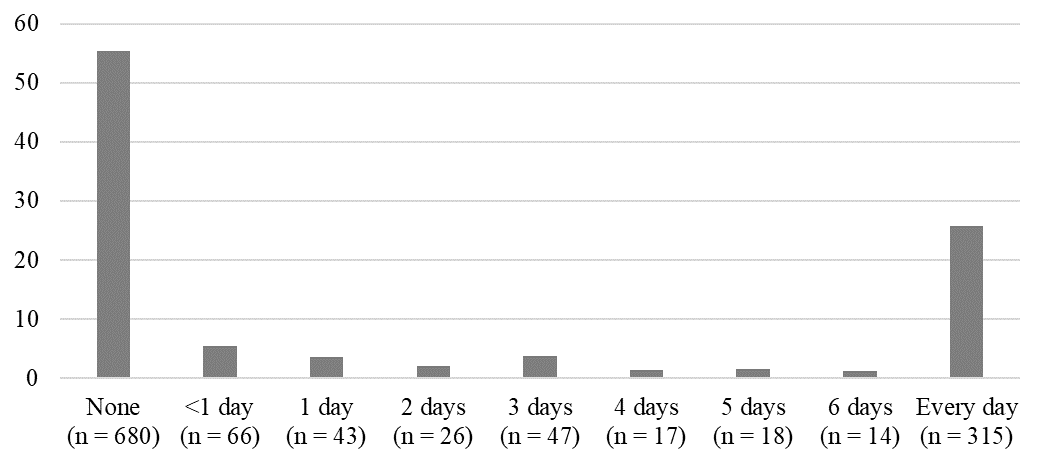


(%)

Daily drinking frequency (per week)

**Additional file 1: Figure S1**. Details for daily drinking frequency.
