# Supplementary material for: Alcohol drinking patterns have a positive association with cognitive function among older people: a cross-sectional study
Source: BMC Geriatr. 2022 Feb 28;22:158. doi: 10.1186/s12877-022-02852-8 (PMC8883620; doi:10.1186/s12877-022-02852-8)
Supplement: Supplementary file 2 — Additional file 2: Table S1. Comparison of characteristics relative to daily alcohol intake. [file 12877_2022_2852_MOESM2_ESM.docx]

| Additional file 2: Table S1. Comparison of characteristics relative to daily alcohol intake | | | | | | | | | |
| --- | --- | --- | --- | --- | --- | --- | --- | --- | --- |
|  | Daily alcohol intake | | | | | | | |  |
|  | None | | Moderate | | Moderate to Excessive | | Excessive | |  |
| Characteristic | (n = 680) | | (n = 424) | | (n = 71) | | (n = 44) | | *p*-value |
| Age: 76 aged group, n (%) | 385 | (56.6) | 262 | (61.8) | 55 | (77.5) | 36 | (81.8) | <0.01 |
| Sex: Men, n (%) | 220 | (32.4) | 290 | (68.4) | 52 | (73.2) | 31 | (70.5) | <0.01 |
| Daily drinking frequency, n (%) |  |  |  |  |  |  |  |  | <0.01 |
| None/week | 680 | (100.0) | 0 | (0.0) | 0 | (0.0) | 0 | (0.0) |  |
| <1 day/week | 0 | (0.0) | 58 | (13.7) | 3 | (4.2) | 1 | (2.3) |  |
| 1–6 days/week | 0 | (0.0) | 136 | (32.1) | 17 | (23.9) | 11 | (25.0) |  |
| Everyday/week | 0 | (0.0) | 230 | (54.2) | 51 | (71.8) | 32 | (72.7) |  |
| Non-daily drinking opportunity, n (%) | 219 | (32.5) | 293 | (70.4) | 59 | (83.1) | 32 | (76.2) | <0.01 |
| Beverage type, n (%) |  |  |  |  |  |  |  |  |  |
| Beer | 0 | (0.0) | 238 | (56.1) | 35 | (49.3) | 25 | (56.8) | <0.01 |
| Japanese spirits | 0 | (0.0) | 104 | (24.5) | 29 | (40.8) | 28 | (63.6) | <0.01 |
| Sake | 0 | (0.0) | 89 | (21.0) | 31 | (43.7) | 12 | (27.3) | <0.01 |
| Wine | 0 | (0.0) | 41 | (9.7) | 6 | (8.5) | 7 | (15.9) | <0.01^†^ |
| Whisky | 0 | (0.0) | 16 | (3.8) | 10 | (14.1) | 6 | (13.6) | <0.01^†^ |
| Current smoking, n (%) | 35 | (5.2) | 31 | (7.3) | 6 | (8.6) | 4 | (9.1) | 0.32^†^ |
| Stroke, n (%) | 63 | (9.3) | 43 | (10.2) | 7 | (9.9) | 4 | (9.1) | 0.97 |
| Hypertension, n (%) | 486 | (72.1) | 313 | (74.3) | 54 | (76.1) | 34 | (77.3) | 0.72 |
| Diabetes mellitus, n (%) | 123 | (18.7) | 78 | (18.9) | 9 | (12.9) | 4 | (9.3) | 0.24 |
| Dyslipidemia, n (%) | 474 | (70.9) | 230 | (55.8) | 41 | (58.6) | 25 | (58.1) | <0.01 |
| Atherosclerosis, n (%) | 536 | (78.8) | 363 | (85.8) | 56 | (78.9) | 34 | (77.3) | <0.05 |
| WHO-5-J (≥ 13), n (%) | 542 | (79.9) | 323 | (76.5) | 59 | (84.3) | 34 | (77.3) | 0.37 |
| Living alone, n (%) | 197 | (29.3) | 65 | (15.4) | 16 | (22.9) | 10 | (22.7) | <0.01 |
| Frequency of going out, n (%) |  |  |  |  |  |  |  |  | 0.31 |
| <1 time/week | 43 | (6.4) | 34 | (8.1) | 7 | (5.6) | 0 | (0.0) |  |
| 1–2 times/week | 111 | (16.4) | 69 | (16.4) | 7 | (9.9) | 4 | (9.1) |  |
| 3–4 times/week | 154 | (22.8) | 97 | (23.0) | 16 | (22.5) | 9 | (20.5) |  |
| 5–6 times/week | 135 | (20.0) | 69 | (16.4) | 15 | (21.1) | 8 | (18.2) |  |
| Every day | 232 | (34.4) | 153 | (36.3) | 29 | (40.8) | 23 | (52.3) |  |
| Education, n (%) |  |  |  |  |  |  |  |  | <0.01 |
| ≤9 years | 169 | (24.9) | 100 | (23.6) | 17 | (23.9) | 7 | (15.9) |  |
| 10–12 years | 340 | (50.1) | 187 | (44.1) | 28 | (39.4) | 15 | (34.1) |  |
| ≥13 years | 169 | (24.9) | 137 | (32.3) | 26 | (36.6) | 22 | (50.0) |  |
| Economic status, n (%) |  |  |  |  |  |  |  |  | 0.26 |
| Not satisfied | 129 | (19.1) | 64 | (15.2) | 18 | (25.4) | 9 | (20.5) |  |
| Neutral | 405 | (60.0) | 272 | (64.5) | 43 | (60.6) | 24 | (54.5) |  |
| Satisfied | 141 | (20.9) | 86 | (20.4) | 10 | (14.1) | 11 | (25.0) |  |
| MoCA-J score, mean (SD) | 22.6 | (4.0) | 22.9 | (3.8) | 22.7 | (3.5) | 23.4 | (3.1) | 0.37 |
| Notes: 76 and 86 aged groups included subjects 75–77 and 85–87 years old, respectively. The criteria for alcohol intake were defined as follows. For men, “Moderate” was >0 g and <40 g, “Moderate to Excessive” was ≥40 g and <60 g, and “Excessive” was ≥60 g. For women, the threshold values used were half as high as those used for men. | | | | | | | | | |
| Abbreviations: SD, standard deviation; WHO-5-J, Japanese version of the WHO Five Well-Being Index; MoCA-J, Japanese version of the Montreal Cognitive Assessment. | | | | | | | | | |
| *p*-values were based on chi-square tests for categorical variables and analysis of variance for continuous variables.  ^†^*p*-values were based on Fisher’s Exact test. | | | | | | | | | |
