# Supplementary material for: Alcohol drinking patterns have a positive association with cognitive function among older people: a cross-sectional study
Source: BMC Geriatr. 2022 Feb 28;22:158. doi: 10.1186/s12877-022-02852-8 (PMC8883620; doi:10.1186/s12877-022-02852-8)
Supplement: Supplementary file 3 — Additional file 3: Table S2. Comparison of characteristics relative to wine consumption. [file 12877_2022_2852_MOESM3_ESM.docx]

| Additional file 3: Table S2. Comparison of characteristics relative to wine consumption | | | | | |
| --- | --- | --- | --- | --- | --- |
|  | Wine | | | |  |
|  | Non-drinker | | Drinker | |  |
| Characteristic | (n = 1172) | | (n = 54) | | *p*-value |
| Age: 76 aged group, n (%) | 704 | (60.1) | 39 | (72.2) | 0.07 |
| Sex: Men, n (%) | 571 | (48.7) | 23 | (42.6) | 0.38 |
| Daily drinking frequency, n (%) |  |  |  |  | <0.01 |
| None/week | 680 | (58.0) | 0 | (0.0) |  |
| <1 day/week | 56 | (4.8) | 10 | (18.5) |  |
| 1–6 days/week | 141 | (12.0) | 24 | (44.4) |  |
| Everyday/week | 295 | (25.2) | 20 | (37.0) |  |
| Daily alcohol intake, n (%) |  |  |  |  | <0.01^†^ |
| None | 680 | (58.4) | 0 | (0.0) |  |
| Moderate | 383 | (32.9) | 41 | (75.9) |  |
| Moderate to Excessive | 65 | (5.6) | 6 | (11.1) |  |
| Excessive | 37 | (3.2) | 7 | (13.0) |  |
| Non-daily drinking opportunity, n (%) | 571 | (49.4) | 35 | (64.8) | <0.05 |
| Beverage type, n (%) |  |  |  |  |  |
| Beer | 276 | (23.5) | 22 | (40.7) | <0.01 |
| Japanese spirits | 156 | (13.3) | 5 | (9.3) | 0.39 |
| Sake | 128 | (10.9) | 4 | (7.4) | 0.42 |
| Whisky | 28 | (2.4) | 4 | (7.4) | <0.05^†^ |
| Current smoking, n (%) | 77 | (6.6) | 0 | (0.0) | 0.08^†^ |
| Stroke, n (%) | 110 | (9.4) | 7 | (13.0) | 0.38 |
| Hypertension, n (%) | 835 | (73.3) | 39 | (72.2) | 0.86 |
| Diabetes mellitus, n (%) | 205 | (18.0) | 9 | (16.7) | 0.80 |
| Dyslipidemia, n (%) | 741 | (64.6) | 33 | (61.1) | 0.60 |
| Atherosclerosis, n (%) | 956 | (81.6) | 39 | (72.2) | 0.08 |
| WHO-5-J (≥13), n (%) | 919 | (78.7) | 43 | (79.6) | 0.88 |
| Living alone, n (%) | 275 | (23.7) | 13 | (24.1) | 0.95 |
| Frequency of going out, n (%) |  |  |  |  | 0.57 |
| <1 time/week | 80 | (6.9) | 2 | (3.7) |  |
| 1–2 times/week | 183 | (15.7) | 8 | (14.8) |  |
| 3–4 times/week | 263 | (22.6) | 15 | (27.8) |  |
| 5–6 times/week | 215 | (18.5) | 13 | (24.1) |  |
| Every day | 424 | (36.4) | 16 | (29.6) |  |
| Education, n (%) |  |  |  |  | <0.01 |
| ≤9 years | 287 | (24.5) | 7 | (13.0) |  |
| 10–12 years | 559 | (47.8) | 17 | (31.5) |  |
| ≥13 years | 324 | (27.7) | 30 | (55.6) |  |
| Economic status, n (%) |  |  |  |  | 0.38 |
| Not satisfied | 214 | (18.4) | 6 | (11.1) |  |
| Neutral | 713 | (61.2) | 35 | (64.8) |  |
| Satisfied | 238 | (20.4) | 13 | (24.1) |  |
| MoCA-J score, mean (SD) | 22.6 | (3.9) | 24.4 | (3.5) | <0.01 |
| Notes: 76 and 86 aged groups included subjects 75-77 and 85-87 years old, respectively. The criteria for alcohol intake were defined as follows. For men, “Moderate” was >0 g and <40 g, “Moderate to Excessive” was ≥40 g and <60 g, and “Excessive” was ≥60 g. For women, the threshold values used were half as high as those used for men. | | | | | |
| Abbreviations: SD, standard deviation; WHO-5-J, Japanese version of the WHO Five Well-Being Index; MoCA-J, Japanese version of the Montreal Cognitive Assessment. | | | | | |
| *p*-values were based on chi-square tests for categorical variables and analysis of variance for continuous variables.  ^†^*p*-values were based on Fisher’s Exact test. | | | | | |
