# Supplementary material for: Alcohol drinking patterns have a positive association with cognitive function among older people: a cross-sectional study
Source: BMC Geriatr. 2022 Feb 28;22:158. doi: 10.1186/s12877-022-02852-8 (PMC8883620; doi:10.1186/s12877-022-02852-8)
Supplement: Supplementary file 4 — Additional file 4: Table S3. Comparison of characteristics relative to non-daily drinking opportunity. [file 12877_2022_2852_MOESM4_ESM.docx]

| Additional file 4: Table S3. Comparison of characteristics relative to non-daily drinking opportunity | | | | | |
| --- | --- | --- | --- | --- | --- |
|  | Non-daily drinking opportunity | | | |  |
|  | No | | Yes | |  |
| Characteristic | (n = 603) | | (n = 606) | | *p*-value |
| Age: 76 aged group, n (%) | 303 | (55.2) | 405 | (66.8) | <0.01 |
| Sex: Men, n (%) | 229 | (38.0) | 359 | (59.2) | <0.01 |
| Daily drinking frequency, n (%) |  |  |  |  | <0.01 |
| None/week | 454 | (75.3) | 219 | (36.1) |  |
| <1 day/week | 25 | (4.1) | 41 | (6.8) |  |
| 1–6 days/week | 47 | (7.8) | 117 | (19.3) |  |
| Everyday/week | 77 | (12.8) | 229 | (37.8) |  |
| Daily alcohol intake, n (%) |  |  |  |  | <0.01 |
| None | 454 | (75.8) | 219 | (36.3) |  |
| Moderate | 123 | (20.5) | 293 | (48.6) |  |
| Moderate to Excessive | 12 | (2.0) | 59 | (9.8) |  |
| Excessive | 10 | (1.7) | 32 | (5.3) |  |
| Beverage type, n (%) |  |  |  |  |  |
| Beer | 75 | (12.4) | 215 | (35.5) | <0.01 |
| Japanese spirits | 34 | (5.6) | 124 | (20.5) | <0.01 |
| Sake | 34 | (5.6) | 97 | (16.0) | <0.01 |
| Wine | 19 | (3.2) | 35 | (5.8) | <0.05 |
| Whisky | 6 | (1.0) | 25 | (4.1) | <0.01 |
| Current smoking, n (%) | 33 | (5.5) | 44 | (7.3) | 0.19 |
| Stroke, n (%) | 63 | (10.4) | 53 | (8.8) | 0.32 |
| Hypertension, n (%) | 433 | (72.5) | 447 | (74.1) | 0.53 |
| Diabetes mellitus, n (%) | 111 | (19.2) | 102 | (17.1) | 0.36 |
| Dyslipidemia, n (%) | 393 | (66.7) | 372 | (62.5) | 0.13 |
| Atherosclerosis, n (%) | 498 | (82.6) | 482 | (79.7) | 0.19 |
| WHO-5-J (≥13), n (%) | 460 | (76.4) | 487 | (80.9) | 0.06 |
| Living alone, n (%) | 162 | (27.0) | 122 | (20.4) | <0.01 |
| Frequency of going out, n (%) |  |  |  |  | 0.24 |
| <1 time/week | 41 | (6.8) | 40 | (6.7) |  |
| 1–2 times/week | 107 | (17.8) | 82 | (13.6) |  |
| 3–4 times/week | 141 | (23.5) | 132 | (22.0) |  |
| 5–6 times/week | 108 | (18.0) | 119 | (19.8) |  |
| Every day | 204 | (33.9) | 228 | (37.9) |  |
| Education, n (%) |  |  |  |  | 0.37 |
| ≤9 years | 151 | (25.1) | 139 | (23.0) |  |
| 10–12 years | 288 | (47.8) | 281 | (46.4) |  |
| ≥13 years | 163 | (27.1) | 185 | (30.6) |  |
| Economic status, n (%) |  |  |  |  | 0.51 |
| Not satisfied | 117 | (19.5) | 102 | (17.0) |  |
| Neutral | 362 | (60.2) | 377 | (62.7) |  |
| Satisfied | 122 | (20.3) | 122 | (20.3) |  |
| MoCA-J score, mean (SD) | 22.3 | (4.3) | 23.2 | (3.5) | <0.01 |
| Notes: 76 and 86 aged groups included subjects 75–77 and 85–87 years old, respectively. The criteria for alcohol intake were defined as follows. For men, “Moderate” was >0 g and <40 g, “Moderate to Excessive” was ≥40 g and <60 g, and “Excessive” was ≥60 g. For women, the threshold values used were half as high as those used for men. | | | | | |
| Abbreviations: SD, standard deviation; WHO-5-J, Japanese version of the WHO Five Well-Being Index; MoCA-J, Japanese version of the Montreal Cognitive Assessment. | | | | | |
| *p*-values were based on chi-square tests for categorical variables and analysis of variance for continuous variables. | | | | | |
