# Supplementary material for: Alcohol drinking patterns have a positive association with cognitive function among older people: a cross-sectional study
Source: BMC Geriatr. 2022 Feb 28;22:158. doi: 10.1186/s12877-022-02852-8 (PMC8883620; doi:10.1186/s12877-022-02852-8)
Supplement: Supplementary file 5 — Additional file 5: Table S4. Comparison of characteristics relative to sex. [file 12877_2022_2852_MOESM5_ESM.docx]

| Additional file 5: Table S4. Comparison of characteristics relative to sex | | | | | |
| --- | --- | --- | --- | --- | --- |
|  | Sex | | | |  |
|  | Men | | Women | |  |
| Characteristic | (n = 594) | | (n = 632) | | *p*-value |
| Age: 76 aged group, n (%) | 346 | (58.2) | 397 | (62.8) | 0.10 |
| Daily drinking frequency, n (%) |  |  |  |  | <0.01 |
| None/week | 220 | (37.0) | 460 | (72.8) |  |
| <1 day/week | 29 | (4.9) | 37 | (5.9) |  |
| 1–6 days/week | 94 | (15.8) | 71 | (11.2) |  |
| Everyday/week | 251 | (42.3) | 64 | (10.1) |  |
| Daily alcohol intake, n (%) |  |  |  |  | <0.01 |
| None | 220 | (37.1) | 460 | (73.5) |  |
| Moderate | 290 | (48.9) | 134 | (21.4) |  |
| Moderate to Excessive | 52 | (8.8) | 19 | (3.0) |  |
| Excessive | 31 | (5.2) | 13 | (2.1) |  |
| Non-daily drinking opportunity, n (%) | 359 | (61.1) | 247 | (39.8) | <0.01 |
| Beverage type, n (%) |  |  |  |  |  |
| Beer | 199 | (33.5) | 99 | (15.7) | <0.01 |
| Japanese spirits | 139 | (23.4) | 22 | (3.5) | <0.01 |
| Sake | 107 | (18.0) | 25 | (4.0) | <0.01 |
| Wine | 23 | (3.9) | 31 | (4.9) | 0.38 |
| Whisky | 29 | (4.9) | 3 | (0.5) | <0.01 |
| Current smoking, n (%) | 63 | (10.7) | 14 | (2.2) | <0.01 |
| Stroke, n (%) | 78 | (13.2) | 39 | (6.2) | <0.01 |
| Hypertension, n (%) | 439 | (74.4) | 453 | (72.2) | 0.40 |
| Diabetes mellitus, n (%) | 128 | (22.1) | 86 | (14.0) | <0.01 |
| Dyslipidemia, n (%) | 336 | (58.1) | 438 | (70.3) | <0.01 |
| Atherosclerosis, n (%) | 526 | (88.7) | 469 | (74.2) | <0.01 |
| WHO-5-J (≥ 13), n (%) | 462 | (78.2) | 500 | (79.4) | 0.61 |
| Living alone, n (%) | 75 | (12.8) | 21.3 | (33.9) | <0.01 |
| Frequency of going out, n (%) |  |  |  |  | 0.06 |
| <1 time/week | 49 | (8.3) | 33 | (5.2) |  |
| 1–2 times/week | 92 | (15.6) | 99 | (15.7) |  |
| 3–4 times/week | 121 | (20.6) | 157 | (24.9) |  |
| 5–6 times/week | 102 | (17.3) | 126 | (20.0) |  |
| Every day | 224 | (38.1) | 216 | (34.2) |  |
| Education, n (%) |  |  |  |  | <0.01 |
| ≤9 years | 140 | (23.6) | 154 | (24.4) |  |
| 10–12 years | 226 | (38.1) | 350 | (55.5) |  |
| ≥13 years | 227 | (38.3) | 127 | (20.1) |  |
| Economic status, n (%) |  |  |  |  | 0.43 |
| Not satisfied | 106 | (18.0) | 114 | (18.1) |  |
| Neutral | 352 | (59.9) | 396 | (62.8) |  |
| Satisfied | 130 | (22.1) | 121 | (19.2) |  |
| MoCA-J score, mean (SD) | 22.3 | (3.9) | 23.1 | (3.9) | <0.01 |
| Notes: 76 and 86 aged groups included subjects 75-77 and 85-87 years old, respectively. The criteria for alcohol intake were defined as follows. For men, “Moderate” was >0 g and <40 g, “Moderate to Excessive” was ≥40 g and <60 g, and “Excessive” was ≥60 g. For women, the threshold values used were half as high as those used for men. | | | | | |
| Abbreviations: SD, standard deviation; WHO-5-J, Japanese version of the WHO Five Well-Being Index; MoCA-J, Japanese version of the Montreal Cognitive Assessment. | | | | | |
| *p*-values were based on chi-square tests for categorical variables and analysis of variance for continuous variables. | | | | | |
